# Analytical sensitivity of current best-in-class malaria rapid diagnostic tests

**DOI:** 10.1186/s12936-017-1780-5

**Published:** 2017-03-24

**Authors:** Alfons Jimenez, Roxanne R. Rees-Channer, Rushini Perera, Dionicia Gamboa, Peter L. Chiodini, Iveth J. González, Alfredo Mayor, Xavier C. Ding

**Affiliations:** 1 ISGlobal, Barcelona Ctr. Int. Health Res. (CRESIB), Hospital Clínic-Universitat de Barcelona, Barcelona, Spain; 2 Hospital for Tropical Diseases, University College London Hospitals NHS Foundation Trust, London, UK; 3 Departamento de Ciencias Celulares y Moleculares, Facultad de Ciencias y Filosofía & Instituto de Medicina Tropical Alexander Von Humboldt, Universidad Peruana Cayetano Heredia, Lima, Peru; 4 Centro de Investigação em Saúde da Manhiça (CISM), Maputo, Mozambique; 5 FIND, Geneva, Switzerland

**Keywords:** Malaria rapid diagnostic test, HRP2, pLDH, Analytical sensitivity

## Abstract

**Background:**

Rapid diagnostic tests (RDTs) are today the most widely used method for malaria diagnosis and are recommended, alongside microscopy, for the confirmation of suspected cases before the administration of anti-malarial treatment. The diagnostic performance of RDTs, as compared to microscopy or PCR is well described but the actual analytical sensitivity of current best-in-class tests is poorly documented. This value is however a key performance indicator and a benchmark value needed to developed new RDTs of improved sensitivity.

**Methods:**

Thirteen RDTs detecting either the *Plasmodium falciparum* histidine rich protein 2 (HRP2) or the plasmodial lactate dehydrogenase (pLDH) antigens were selected from the best performing RDTs according to the WHO–FIND product testing programme. The analytical sensitivity of these products was evaluated using a range of reference materials including *P. falciparum* and *Plasmodium vivax* whole parasite samples as well as recombinant proteins.

**Results:**

The best performing HRP2-based RDTs could detect all *P. falciparum* cultured samples at concentrations as low as 0.8 ng/mL of HRP2. The limit of detection of the best performing pLDH-based RDT specifically detecting *P. vivax* was 25 ng/mL of pLDH.

**Conclusion:**

The analytical sensitivity of *P. vivax* and Pan pLDH-based RDTs appears to vary considerably from product to product, and improvement of the limit-of-detection for *P. vivax* detecting RDTs is needed to match the performance of HRP2 and Pf pLDH-based RDTs for *P. falciparum*. Different assays using different reference materials produce different values for antigen concentration in a given specimen, highlighting the need to establish universal reference assays.

**Electronic supplementary material:**

The online version of this article (doi:10.1186/s12936-017-1780-5) contains supplementary material, which is available to authorized users.

## Background

The development of point-of-care lateral flow immunochromatographic assays in the mid 1990s for the detection of malaria parasites in minute amounts of capillary blood has radically changed the diagnosis of this parasitic disease in endemic areas. These tests, commonly referred to as rapid diagnostic tests (RDTs), have established themselves as an extremely valuable alternative to the examination of stained blood smears by light microscopy. While light microscopy remains a method of choice for malaria diagnosis in many areas, the use of quality-controlled RDTs is considered adequate and recommended by the World Health Organization (WHO) for the parasitological confirmation of suspected malaria cases [1]. With approximately 314 million units sold in 2014, malaria RDTs represent a major commodity in the fight against malaria and the primary method for malaria diagnosis in comparison to the estimated 203 million suspected cases tested by microscopy worldwide in 2014 [2].

RDTs are affordable, with retail prices varying between 0.2 and 1.0 US dollar and an estimated cost of diagnosis between 1.0 and 2.0 US dollars [3, 4]. Other key advantages are their simplicity, which enables their use in a point-of-care mode by minimally trained individuals, and the rapidity with which test results can be obtained, typically within 20 min or less. Light microscopy is not necessarily much more expensive, with a cost of diagnosis estimated between 1.0 and 2.0 US dollars [3]. However, it is more complex to implement and to maintain at a good quality level, requiring a microscope and a laboratory to stain and read blood smears, and is critically dependent on the training and performance level of the microscopist to generate accurate results.

Malaria RDTs typically consist of a plastic cassette enclosing a nitrocellulose membrane strip, at the bottom of which are placed lysing agents and dye-labelled antibodies specifically recognizing a *Plasmodium* antigen of interest. Upon the addition of blood (typically 10 µL or less) and buffer to the bottom of the strip, the red blood cells lyse, mix with the labelled antibodies, and migrate along the membrane strip by capillarity toward a fine line of bound antibodies. If the antigen of interest is present in the investigated blood sample at a sufficiently high concentration, the antigen-labelled antibody complexes will be captured on this line and the accumulated dye will become visible to the naked eye. A control line, coated with either the antigen of interest or antibodies recognizing directly the labelled antibody, is also typically included to assess the integrity of individual RDT reagents and their correct diffusion across the nitrocellulose membrane.

Current commercial malaria RDTs target one or more of three standard *Plasmodium* proteins. These are histidine rich protein 2 (HRP2) and two enzymes of the *Plasmodium* glycolytic pathway: aldolase and plasmodial lactate dehydrogenase (pLDH). HRP2 is specific to *Plasmodium falciparum* whereas aldolase and pLDH are expressed in all five human-infecting *Plasmodium* spp. and allow, in principle, the detection of all of them (pan-RDT). In addition, and because pLDH is not fully conserved across *Plasmodium* species, the selection of species-specific epitopes has allowed the development of antibodies recognizing specifically *P. falciparum* pLDH (Pf-pLDH), *Plasmodium vivax* pLDH (Pv-pLDH) or collectively *P. vivax*, *Plasmodium ovale*, and *Plasmodium malariae* pLDH (Pvom-pLDH). HRP2-based RDTs are the main product type for the detection of *P. falciparum*, while species-specific detection of *P. vivax*, the second most prevalent malaria species in humans, requires the use of Pv-pLDH-based RDTs.

The performance of RDTs is very often analysed from the clinical point-of-view: the capacity of RDTs to identify correctly malaria positive and negative samples is evaluated in comparison to a reference method, such as light microscopy or PCR, and diagnostic sensitivity and specificity values are reported with associated confidence intervals [5, 6]. Little is known however, about the analytical performance of RDTs, especially the analytical sensitivity, which corresponds to the lowest detectable concentration of the target analyte. This parameter is especially important as it directly relates to the capacity of malaria RDTs to detect and correctly diagnose malaria parasites early during the course of an infection, which is required rapidly to alleviate symptoms and prevent as much as possible, the appearance of gametocytes and the transmission of parasites. The analytical sensitivity of current RDTs is also a key benchmark value when considering the development of RDTs with improved sensitivity, which might enable the diagnosis of not only clinical cases of malaria but also low density and asymptomatic infections [7], which are currently considered to be beyond the detection limit of standard malaria RDTs.

In this study, the analytical sensitivity of 13 RDTs selected amongst the best performing ones were evaluated using a range of reference materials calibrated for their HRP2 or pLDH content. The limits of detection, expressed in target analyte concentrations, for these products are reported and the implications of these values on the performance on malaria RDTs are discussed.

## Methods

### Rapid diagnostic tests selection

The selection of the best-in-class RDTs was based on the results of the WHO–FIND product testing programme (Rounds 1–6), which has evaluated the performance of 171 unique RDT products on malaria samples at standardized parasitaemia (200 and 2000 parasites/µL) [8]. The reactivity of each RDT against *P. falciparum* or *P. vivax* isolates is reported as a panel detection score (PDS) which reflects the percentage of positive samples correctly detected when tested in duplicate by two distinct lots. RDTs are also tested against a panel of clean negative samples from which a false-positive rate is derived. Finally, the percentage of invalid results is also available.

The following selection criteria were used to identify the best-in-class HRP2 and pLDH (Pv-pLDH, Pf-pLDH, Pvom-pLDH and Pan-pLDH) products. For HRP2-based RDTs, a PDS ≥ 85% for *P. falciparum* samples at 200 p/µL and a false positivity rate <0.5% was required. For pLDH-based RDTs, the selection criteria were not as stringent as for HRP2-based RDTs, because of the lower number of existing products and the overall lower performance of RDTs detecting this antigen. For each type of pLDH-detecting RDT, a PDS ≥ 75% for *P. falciparum* or *P. vivax* samples at 200 p/µL and a false positivity rate <5% was required. When products with identical performance were available, the selection was made to maximize the diversity of product manufacturers. The identity of the selected RDTs was anonymized by numbering 1–13 as the goal of this study was not to evaluate the performance of specific products or manufacturers but to report the analytical sensitivity of current best-in-class RDTs.

### HRP2 reference materials

*Plasmodium falciparum* culture samples from three laboratory strains and a recombinant HRP2 protein expressed in *Escherichia coli* (Microcoat GmbH, Germany, lot number ESS_1426, manufactured July 2014) based on the HRP2 sequence of the W2 *P. falciparum* strain, were selected as HRP2 reference materials. The Benin I, Santa Lucia, and PH1 strains were cultured under standard hypoxic conditions as previously reported [9]. Cultures in exponential growth phase were harvested, infected red blood cells were spun down, aliquoted, and frozen at −80 °C for long term storage. The HRP2 concentrations contained within the cultured samples were measured by a commercially available ELISA (see below) and twofold serial dilutions were prepared using malaria negative whole blood to obtain samples at the following HRP2 concentrations: 3.2, 1.6, 0.8, 0.4, 0.2, 0.1, 0.05 and 0.025 ng/mL.

### pLDH reference materials

*Plasmodium falciparum* culture samples, *P. vivax* isolates from malaria patients (to circumvent the absence of *P. vivax* culture system), as well as *P. falciparum* and *P. vivax* recombinant pLDH proteins were used as reference materials. The FCQ79, W2, and PH1 *P. falciparum* strains were cultured under standard hypoxic conditions (of note these partially differ from the strains selected for the evaluation of HRP2 RDTs because of the limited strain availability at the respective laboratories where the two types of RDTs were evaluated). Cultures in exponential growth phase were harvested, infected red blood cells were spun down, aliquoted, and frozen at −80 °C for long term storage. *Plasmodium vivax* isolates were collected from symptomatic adult volunteers with a *P. vivax* mono-species infection as confirmed by microscopy during a specimen collection campaign organized in April 2016 in the area of Iquitos (Peru). The study protocol was approved by the institutional review board the Universidad Peruana Cayetano Heredia (Lima, Peru).

A volume of venous whole blood was collected, anticoagulated using EDTA, aliquoted, and frozen within 24 h at −80 °C for long term storage. Five samples, referred to here as *Pv1*–*Pv5*, were selected for this study and confirmed to be *P. vivax* mono-species infections by nested PCR, according to a previously published protocol [10]. Purified recombinant *P. falciparum* and *P. vivax* pLDH proteins expressed in *E. coli* were obtained from MyBioSource (USA, ref. MBS319810 and MBS319848, respectively) and are referred here as Pf-pLDH EC and Pv-pLDH EC. Purified recombinant *P. falciparum* and *P. vivax* pLDH proteins expressed in insect cells were produced by ReliaTech GmbH (Germany) and are referred here as Pf-pLDH EUK and Pv-pLDH EUK. The pLDH concentrations of the culture samples, field isolates and recombinant proteins were measured by a commercially available ELISA (see below) to prepare serial dilutions using malaria negative whole blood. Ten-fold serial dilutions ranging from 5000 to 0.5 ng/mL were prepared for an initial evaluation of the RDT analytical sensitivities. Additional twofold serial dilutions, with five dilutions each, centred around the 10-fold dilution values, were further prepared to obtain more precise estimates of the RDT analytical sensitivities. Series ranged from 2000 to 125 ng/mL, 200 to 12.5 ng/mL, 20 to 1.25 ng/mL, and 2 to 0.125 ng/mL.

### HRP2 quantitative ELISA

The quantification of HRP2 content within reference materials was done using the Malaria Ag Pf ELISA kit (ref. 05EK50) manufactured by Standard Diagnostics (South Korea). ELISAs were performed as recommended by the manufacturer, with minor modifications to improve sensitivity. The sample incubation step was performed at 37 °C with agitation at 600 rpm using a 96 well multi-plate thermo-shaker (PHMP4, Grant-bio) and substrate development time increased from 10 to 20 min. Absorbances at 450 and 620 nm were read using a Microtek DS dynamic microplate spectrophotometer (Bio-Tek).

A purified HRP2 recombinant protein expressed in *E. coli* (Microcoat GmbH, Germany, lot ESS_1426, manufactured July 2014) based on the allele of the W2 *P. falciparum* laboratory strain (type B) of known concentration, as determined by absorbance at 280 nm, was used as reference standard. Briefly, eight point serial dilution standard curves were tested in quadruplicate and the average absorbance values were used to generate both arithmetic and logarithmic calibration curves in Excel 2010 (Microsoft, version 14.0.7106.5003). Selected points from the logarithmic and arithmetic curves were used to plot straight line graphs and the applicable trend line equations used to calculate HRP2 concentrations from mean optical density (OD) readings recorded for each test sample. Each test sample of unknown concentration was assessed in duplicate at three serial twofold dilutions, and the OD values for each dilution were averaged to obtain the mean OD. The resulting calculated sample HRP2 concentrations were then multiplied out by the applicable dilution factors and these three final concentration values averaged again to give a final HRP2 concentration for each test sample. Samples with absorbance values out of the range of the standard curve were re-assayed at adjusted dilutions. Final concentration values were the average of at least three independent assays for all samples of unknown concentrations.

### pLDH quantitative ELISA

The quantification of the pLDH content within reference materials was done using the Qualisa Malaria kit (ref. 40903480) from Qualpro Diagnostics (India). This kit is an ELISA test based on the quantification of pLDH samples with immobilized pan-specific anti-pLDH capture monoclonal antibody on 96-well plates that can be used for field and cultured *Plasmodium* spp. samples or purified recombinant forms of pLDH. ELISAs were performed as recommended by the manufacturer, with minor modifications. The antibody reagent and sample diluent were added to the plate wells before the blood samples. The sample incubation step was extended from 30 min to 1 h and the detection step was shortened from 30 to 20 min. Washing steps were performed using an ELx405 Microplate Washer (Bio-Tek). The absorbances at 450 and 620 nm were read using an Epoch Microplate Spectrophotometer EPOCH (Bio-Tek).

A purified Pv-pLDH recombinant protein prepared by MicroMol GmbH (Germany) of known concentration, as determined by absorbance at 280 nm, was used as a reference standard in the ELISA. Briefly, eight point serial dilution standard curves were tested in triplicate and the average absorbance values and expected pLDH concentration were used to generate a five-parameter logistic equation using GraphPad Prism 6 (GraphPad Software). Each test sample of unknown concentration was assessed in duplicate at three serial twofold dilutions, and the OD values for each dilution were averaged to obtain the mean OD. The resulting calculated sample pLDH concentrations were then multiplied out by the applicable dilution factors and these three final concentration values averaged again to give a final pLDH concentration for each test sample. Samples with absorbance values out of the range of the standard curve were re-assayed at adjusted dilutions. Final concentration values were the average of at least three independent assays for all samples of unknown concentrations.

### Rapid diagnostic tests evaluation

The reference materials described above and prepared in malaria negative whole blood were used to evaluate the reactivity of the selected RDTs according to the respective manufacturer’s recommendations. For each product, a single lot was sourced directly from the manufacturing company, stored according to recommendations and used before its expiration date. RDTs were always tested in duplicate and the test line colour intensity was recorded according to a visual scale ranging from 0 (no signal) to 4 (strong intensity). An average score above 0 was classified as a positive test result while an average score of 0 was classified as negative. The HRP2-based RDTs were evaluated at the Hospital for Tropical Diseases laboratory (London, England) and the pLDH-based RDTs were evaluated at the Barcelona Institute for Global Health (Barcelona, Spain). At each laboratory, duplicate RDTs were prepared and read by the same technician. A workshop was organized to harmonize the scoring at both laboratories and ensure results comparability.

## Results

### Rapid diagnostic tests selection

Following the pre-defined criteria, five HRP2- and eight pLDH-based RDTs were selected (Table 1, numbered 1–13 according to the antigen of interest and then by decreasing PDS for the relevant antigen). Out of the eight pLDH products selected, three detect Pv-pLDH (RDTs 6, 7, and 8), two detect Pf-pLDH (RDTs 10 and 11), one detects Pvom-pLDH (RDT 9) and three detect Pan-pLDH (RDTs 10, 12, and 13).

**Table 1:** Selected RDTs

RDT	Manufacturer	PDS for *P. falciparum* at 200 p/µL	PDS for *P. vivax* at 200 p/µL	False-positivity rate	Antigen of interest	Additional antigen
1	A	95.0%	n/a	0%	HRP2	–
2	B	95.0%	n/a	0.4%	HRP2	–
3	C	90.8%	94.1%	0%	HRP2	Pv-pLDH
4	D	86.9%	n/a	0%	HRP2	–
5	B	85%	74.3%	0%	HRP2	Pan-pLDH
6	E	92.9	100%	0.5%	Pv-pLDH	HRP2
7	F	79.6%	100%	1.5%	Pv-pLDH	HRP2
8	A	96%	95%	0%	Pv-pLDH	HRP2
9	C	89.8%	91.2%	0.3%	Pvom-pLDH	HRP2
10	C	88.9%	91.4%	1.3%	Pf-PLDH, Pan-pLDH^a^	–
11	A	87.9%	n/a	0%	Pf-pLDH	HRP2
12	G	77%	100%	n/a	Pan-pLDH	–
13	C	90%	94.3%	1.5%	Pan-pLDH	HRP2

^a^The performance of both test lines of this RDT were selected (as per the selection criteria outlined in the “Methods”) for evaluation

Of note, the independent selection of the best-in-class HRP2- and pLDH-based RDTs showed no overlap despite the relatively large number of combination RDTs detecting simultaneously HRP2 and some variants of pLDH that have been evaluated in the WHO-FIND product testing programme (64 out 129 products).

### Analytical sensitivity of best-in-class HRP2-based rapid diagnostic tests

Five HRP2-based RDTs (RDTs 1–5) were evaluated using samples from three *P. falciparum* laboratory strains and one purified HRP2 recombinant protein. The lowest HRP2 concentrations detected from the twofold serial dilutions are reported in Table 2 for each combination. The lowest HRP2 concentration at which all three *P. falciparum* cultured strains tested here could be detected was 0.8 ng/mL for RDT 1, RDT 2, RDT 4, and RDT 5 and 1.6 ng/mL for RDT 3. The overall lowest HRP2 concentration detected was 0.4 ng/mL, as observed for three out of five RDTs when tested with samples from the PH1 strain. Very similar values were observed with the recombinant HRP2 protein, with limits of detection ranging from 0.8 to 1.6 ng/mL.

**Table 2:** Analytical sensitivity of selected HRP2-based RDTs

Sample type	Sample	HRP2				
		RDT 1 (ng/mL)	RDT 2 (ng/mL)	RDT 3 (ng/mL)	RDT 4 (ng/mL)	RDT 5 (ng/mL)
*P. falciparum* culture	Benin I	0.8	0.8	1.6	0.8	0.8
	Santa Lucia	0.8	0.8	1.6	0.8	0.8
	PH1	0.4	0.8	0.8	0.4	0.4
Recombinant protein	rHRP2 (W2)	0.8	1.6	1.6	0.8	0.8

### Analytical sensitivity of best-in-class pLDH-based rapid diagnostic tests

Eight pLDH-based RDTs were tested (RDTs 6–13) on five *P. vivax* isolates, three *P. falciparum* culture strains, as well as on *P. falciparum* and *P. vivax* purified recombinant proteins expressed in *E. coli* (Pf-pLDH EC and Pv-pLDH EC) or insect cells (Pf-pLDH EUK and Pv-pLDH EUK). The lowest pLDH concentrations detected from the twofold serial dilutions are reported in Table 3 for each combination.

**Table 3:** Analytical sensitivity of selected pLDH-based RDTs

Sample type	Sample	Pv-pLDH			Pvom-pLDH	Pf-pLDH		Pan-pLDH		
		RDT 6 (ng/mL)	RDT 7 (ng/mL)	RDT 8 (ng/mL)	RDT 9 (ng/mL)	RDT 10 (ng/mL)	RDT 11 (ng/mL)	RDT 10 (ng/mL)	RDT 12 (ng/mL)	RDT 13 (ng/mL)
*P. vivax* isolate	*Pv1*	25	25	25	12.5	–	–	500	50	5
	*Pv2*	25	25	25	5	–	–	1000	25	5
	*Pv3*	12.5	25	25	5	–	–	250	25	5
	*Pv4*	25	25	25	10	–	–	500	25	10
	*Pv5*	25	50	25	5	–	–	1000	25	10
*P. vivax* rec. protein	Pv-pLDH EC	10	25	12.5	2.5	*500* ^a^	–	500	25	5
	Pv-pLDH EUK	25	25	25	20	–	–	1000	50	12.5
*P. falciparum* culture	FCQ79	–	–	–	–	2.5	0.5	2.5	50	5
	W2	–	–	–	–	5	1	5	50	10
	PH1	–	–	–	–	2.5	1	2.5	25	5
*P. falciparum* rec. protein	Pf-pLDH EC	*5000* ^a^	–	–	*5000* ^a^	25	10	25	25	10
	Pf-pLDH EUK	–	–	–	–	100	10	50	25	20

A dash indicates a combination for which no reactivity was seen up to the highest concentration tested

^a^False positive results

Pv-pLDH specific RDTs could detect all five *P. vivax* samples at a concentration as low as 50 ng/mL (RDT 7) or 25 ng/mL (RDT 6 and RDT 8). The lowest pLDH concentration detected when testing *P. vivax* samples was 12.5 ng/mL (RDT 6). Very similar values were seen with recombinant pLDH proteins, with both Pv-pLDH proteins showing limits of detection ranging between 10 and 25 ng/mL. The single RDT with a Pvom-pLDH test line (RDT 9) could detect the *P. vivax* samples at concentrations between 5 and 12.5 ng/mL of Pv-pLDH. The limits of detection for the recombinant Pv-pLDH proteins expressed in *E. coli* and insect cells were below and above these values at 2.5 and 20 ng/mL, respectively.

Pf-pLDH RDTs could detect *P. falciparum* culture samples at much lower concentrations compared to Pv-pLDH RDT detecting *P. vivax* samples, with RDT 11 detecting all three *P. falciparum* strains tested at concentrations of 1 ng/mL or lower. RDT 10 detected these samples at concentrations ranging between 2.5 and 5 ng/mL. The limit of detection for recombinant Pf-pLDH proteins was at least one order of magnitude above these values at 100 ng/mL (RDT 10) and 10 ng/mL (RDT 11).

Three RDTs had Pan-pLDH test lines (RDTs 10, 12, and 13). When tested using *P. vivax* reference materials, large differences in the limit of detection were observed. At least 1000 ng/mL was required for RDT 10 to detect all *P. vivax* samples or recombinant proteins, 50 ng/mL for RDT 12 and 12.5 ng/mL for RDT 13. When considering only *P. vivax* samples, RDT 13 could detect all five with a concentration as low as 10 ng/mL. The limits of detection of the *P. falciparum* samples were more consistent between these three RDTs. All three *P. falciparum* culture strains could be detected at 5, 50 and 10 ng/mL by RDT 10, RDT 12, and RDT 13, respectively. For recombinant proteins, it required between 20 and 50 ng/mL to achieve the same.

### Observations on RDT analytical specificities

Species cross-reactivity issues with pLDH-based RDTs have been reported previously in the literature [11]. The purpose of this study was not to directly evaluate this element of the performance of RDTs, yet the serial dilutions of the pLDH reference materials were set at a relatively high initial concentration, 5000 ng/mL, to detect any overt specificity issues.

Reference materials containing whole parasites (i.e. *P. falciparum* cultured samples and *P. vivax* field samples) did not trigger any false positive reaction when tested at concentrations as high as 5000 ng/mL with any pLDH RDT, i.e. *P. falciparum* samples were only detected by Pan-pLDH or Pf-pLDH test lines, while *P. vivax* samples were only detected by Pv-pLDH, Pvom-pLDH and Pan-pLDH test lines. The pLDH recombinant proteins expressed in eukaryotic cells also generated only true positive reactions. In contrast, some false positive reactions were seen with the recombinant pLDH proteins expressed in *E. coli* (Table 3). Pf-pLDH EC at 5000 ng/mL triggered positive reactions on the Pv-pLDH test line of RDT 6 and on the Pvom-pLDH test line of RDT 9. Pv-pLDH EC protein was detected at a concentration as low as 500 ng/mL by the Pf-pLDH test line of RDT 10. More surprisingly, all recombinant pLDH proteins tested, that were expressed in *E. coli* or insect cells, showed some level of cross-reactivity with the HRP2 test lines of the pLDH-based RDTs tested here (Additional file 1: Table S1). While the recombinant pLDH proteins expressed in insect cells were recognized by the HRP2 test lines of RDT 9 and 13, though not at concentrations below 5000 ng/mL, recombinant pLDH proteins expressed in *E. coli* were detected by HRP2 test lines at concentrations as low as 50 ng/mL by some RDTs (RDT 8, RDT 9 and RDT 13).

## Discussion

In this study, the analytical sensitivity of 13 malaria RDTs classified amongst the best performing products according to the results of the WHO–FIND product testing programme is reported [8]. A number of factors directly influence the performance of RDTs, including the antibody characteristics and stability, the antibody immobilization technique, the nitrocellulose type and treatment. The values reported here are expressed in target analyte concentration and not parasite density to provide a direct measurement of the limit of detection of current best RDTs. While blood stage malaria infections are normally characterized by a parasitaemia expressed typically in percentage of parasitized red blood cells or in parasites detected per µL of blood, this value is less appropriate for RDTs since these are not detecting parasites themselves but amounts of target analyte produced by these parasites.

Five HRP2-based RDTs and eight pLDH-based RDTs were selected. This study focuses on these two analytes as the vast majority of current RDTs are based on the detection of HRP2 for the identification of *P. falciparum* infections, or on some combination of the pLDH isoforms for the pan or species-specific detection of the human-infecting *Plasmodium* species. Detection of the *P. vivax* pLDH isoform is also currently the only approach specifically to identify a *P. vivax* infection by RDT, which is of importance in areas of *P. vivax* and *P. falciparum* co-endemicity and where the recommended treatment guidelines are not identical for these species.

The HRP2-based RDTs tested here displayed very similar analytical sensitivities, with four out of five RDTs achieving the detection of all three *P. falciparum* strains tested at HRP2 concentrations down to 0.8 ng/mL. Interestingly, four out of five RDTs systematically detected the PH1 strain at a lower HRP2 concentrations than the Benin I and Santa Lucia strains. HRP2 is a polymorphic antigen, characterized by variable copy numbers of specific repetitive motifs and an earlier study suggested that a correlation might exist between the total number of some of these repeats and the capacity of RDTs to detect specific isoforms [12]. Based on this criterion, HRP2 isoforms can be classified into three types, A, B, and C, with the number of these specific elements and the detectability by RDTs being supposedly higher for type C than B and for type B than A. These results appear to be in line with this suggested correlation as PH1, a type C strain, is apparently detected by the RDTs tested here at lower concentrations than Benin I and Santa Lucia samples, which are type A and type B strains, respectively. However, the low number of strains tested here means that further data are required to support this hypothesis, especially as a study evaluating a larger number of *P. falciparum* isolates did not confirm the correlation between the A, B, and C type HRP2 classification and the detectability by RDTs, leaving the relevance of this classification unclear [13].

The relationship between HRP2 blood level and the peripheral parasitaemia of infected individuals is weak. Some studies reported a lack of correlation between these two parameters [14], while others reported limited correlations [15]. For this reason, it is not possible to translate directly the analytical sensitivity of HRP2-based RDTs reported here into a parasite density threshold. The three products with Pan-pLDH test lines showed contrasting results when tested with wild type *P. vivax* samples, with an overall detection limit of 10 ng/mL (RDT 13), 50 ng/mL (RDT 12), and 1000 ng/mL (RDT 10), suggesting that the sensitivity of Pan-pLDH test lines is very much product-dependent. The limit of detection of Pv-pLDH test lines of *P. vivax* isolates was more consistent with two products at 25 ng/mL and the third one at 50 ng/mL and in line with values reported in a previous study [16].

Similar to HRP2, the lack of a robust correlation between pLDH protein concentration and parasitaemia [16] does not allow expression of these results in parasite densities. RDT performance observed in this study using recombinant proteins is globally in line with that observed when using whole parasites from cultured samples or patient isolates that contain native HRP2 or pLDH proteins. One exception, however, is the higher LODs observed when testing Pf-pLDH RDTs with Pf-pLDH *E. coli* and eukaryotic recombinant proteins. Another observation was the false positive results obtained when testing pLDH recombinant proteins expressed in *E. coli*. The fact that these proteins exhibit a higher level of false positive reactions when tested on RDTs designed to detect a different species suggests that proteins expressed in a prokaryotic system might not be representative of native proteins found within whole parasites and that results generated using such proteins should be considered with caution. The fact that cross-reactivity and false positive results were observed only with recombinant proteins and not with samples containing native proteins suggests that these observations are not a cause for concern for the performance of RDTs on clinical samples. This suggest however that the clinical relevance of quality control materials based on purified recombinant protein should be carefully validated.

It is worth noting that the analytical sensitivity values reported in this study have been measured in reference laboratories and these are likely to represent best-case scenarios as any potential degradation due to product transportation and storage in endemic settings or reading errors by end users are avoided here.

There are currently no universal reference assays or calibrators for the quantification of HRP2 and pLDH. For this reason, it is not possible to establish direct comparisons between the values reported here, measured using a specific assay and calibrator combination, with those reported in other studies that have utilized a different set of methodologies. This highlights the need to establish universally applicable reference tools in order to standardize the units by which antigen concentrations are measured in future evaluations.

## Additional file

**Additional file 1.** Analytical sensitivity of the HRP2 test lines of selected pLDH-based RDTs.
