# Supplementary material for: Analytical sensitivity of current best-in-class malaria rapid diagnostic tests
Source: Malar J. 2017 Mar 24;16:128. doi: 10.1186/s12936-017-1780-5 (PMC5366122; doi:10.1186/s12936-017-1780-5)
Supplement: Supplementary file 1 — Additional file 1. Analytical sensitivity of the HRP2 test lines of selected pLDH-based RDTs. [file 12936_2017_1780_MOESM1_ESM.pdf]

Analytical Sensitivity of Current Best-in-Class Malaria Rapid Diagnostic Tests

Supplementary material

Supplementary Table 1. Analytical sensitivity of the HRP2 test lines of selected pLDH-based RDTs

		HRP2					
Sample type	Sample	RDT 6 (ng/mL)	RDT 7 (ng/mL)	RDT 8 (ng/mL)	RDT 9 (ng/mL)	RDT 11 (ng/mL)	RDT 13 (ng/mL)
P. vivax isolate	*Pv1*	-	-	-	-	-	-
	Pv2	-	-	-	-	-	-
	Pv3	-	-	-	-	-	-
	Pv4	-	-	-	-	-	-
	Pv5	-	-	-	-	-	-
P. vivax rec. protein	Pv-pLDH EC	*500**	*500**	*50**	*50**	*500**	*500**
	Pv-pLDH EUK	-	-	-	*5 000**	-	*5 000**
P. falciparum culture	FCQ79	n/a	n/a	n/a	n/a	n/a	n/a
	W2	n/a	n/a	n/a	n/a	n/a	n/a
	PH1	n/a	n/a	n/a	n/a	n/a	n/a
P. falciparum rec. protein	Pf-pLDH EC	-	-	*500**	*5 000**	*5 000**	*50**
	Pf-pLDH EUK	-	-	-	-	-	*5 000**

*False positive results

The mention "n/a" indicates a condition not tested. A dash indicates a combination for which no reactivity was seen up to the highest concentration tested.
